# Forecasting the Incidence of Testis Cancer in Europe from 2012–2035

**Published:** 2017-10

**Authors:** Ali Asghar VALIPOUR, Mahdi MOHAMMADIAN, Abdollah MOHAMMADIAN-HAFSHEJANI, Sarah ISLAMIE FARSANI

**Affiliations:** 1. Dept. of Public Health, School of Public Health, Abadan School of Medical Sciences, Abadan, Iran; 2. Dept. of Public Health, School of Public Health, Dezful University of Medical Sciences, Dezful, Iran; 3. Dept. of Epidemiology and Biostatistics, Isfahan University of Medical Sciences, Isfahan, Iran; 4. Dept. of Epidemiology and Biostatistics, School of Public Health, Tehran University of Medical Sciences, Tehran, Iran; 5. Student's Research Committee, Shahrekord University of Medical Sciences, Shahrekord, Iran

## Dear Editor-in-Chief

Testis Cancer is the greatest common cancer in the 15–39 yr old men and with white Caucasian race. This cancer consists 0.7% of all men’s cancer in the world. In western cultures, the testis cancer is the cause of 1%–1.5% of the men’s cancer and 5 percent of the urinary tract cancer (1). In the past 50 years in the worldwide, the incidence rate of testis cancer has increased trend (2). As a result, the number of incident cases of testis cancer in the world in the year 2008 and 2012 was more than 52000 and 55000 cases, respectively (3). The largest increase in the incidence of testis cancer was observed in Caucasian white race, while the lowest rates were observed in non-white races (4). Therefore, in the year 2012, the highest incidence of testis cancer has occurred in the west (5, 6) and northern Europe (5) while the lowest incidence has occurred in Asia and Africa (1). In this year, the highest and lowest Age-Standardised Incidence Rates (rate per 100000 population at risk) of testis cancer in European countries was observed in Norway (12.2) and Russian Federation (1.7), respectively. Among European countries, there is a 7 times difference in the incidence rate of testis cancer (7).

In GLOBOCAN project, the expected number of new cancer cases in Europe in 2015,..., 2035 is computed by multiplying the age-specific incidence rates estimated for 2012, by the corresponding expected population for 2015,..., 2035 (8). In 2012, the number of 21548 new cases of testis cancer were observed in the European countries, that the number of 20741 cases (96.25%) are in the age group below 65 yr old and 807 cases (03.75%) are in the age group 65 yr old and more. According to the GLOBOCAN Project forecasts, in European countries in the years 2015, 2020, 2025, 2030 and 2035 the number of new testis cancer cases be equal to 21175, 20555, 20091, 19672 and 19206 cases, respectively. In another word, in the years 2015, 2020, 2025, 2030, 2035 compared to the year 2012, the ratio (%) of new cases reduces to 1.73%, 4.60%, 06.76%, 08.70% and 10.88%, respectively. Although, the number of new cases of testis cancer reduces between the years 2012 and 2035, this reduction will be more in the age group below 65 yr and in the age group 65 yr and more the number of cases will increases (Table 1).

**Table 1:** Predict the number of new cases of testicular cancer in European countries from 2012–2035

***Year***	***Predict number of new cancers***			***Demographic change***		
	**Ages < 65 (yr)**	**Ages >= 65**	**Total**	**Ages < 65**	**Ages >= 65**	**Total**
2012	20741	807	21548	-	-	-
2015	20316	859	21175	−425	+52	−373
2020	19616	939	20555	−1125	+132	−993
2025	19061	1030	20091	−1680	+223	−1457
2030	18545	1127	19672	−2196	+320	−1876
2035	18002	1204	19206	−2739	+397	−2342

- Demographic changes were computed for years 2015–2035, based on the number of testis cancer in European countries in 2012 as a reference group.

In European countries between 2012 to 2035, based on GLOBOCAN Project (8) estimates incidence of testis cancer in people less than 65 yr will have declining trend, but in people, 65 yr and older will have rising trend. Probably increasing the proportion of elderly between 2012 to 2035 years is the cause of increasing trend in the incidence of testis cancer in the European countries. However, health policy makers must have planned for regular screening programs with the aim of diagnosis and treatment of testis cancer in the early stages of disease progression. Therefore, with early diagnosis and treatment of patients, in addition to improving the quality of life of patients, we can decline the number of patients that death due to testis cancer.
